# Effects of Peripheral and Intra-hippocampal Administration of Sodium Salicylate on Spatial Learning and Memory of Rats

**Published:** 2012

**Authors:** Leila Azimi, Ali Pourmotabbed, Mohammad Rasool Ghadami, Seyed Ershad Nedaei, Targol Pourmotabbed

**Affiliations:** 1 *Department of Physiology, School of Medicine, Kermanshah University of Medical Sciences, Kermanshah, Iran *; 2 *Department of Neuroscience, School of Advanced Medical Technologies, Tehran University of Medical Sciences, Tehran, Iran*; 3 *Student of Information Technology, Kermanshah University of Technology, Kermanshah, Iran*

**Keywords:** Cyclooxygenase, Rat, Sodium salicylate, Spatial memory

## Abstract

**Objective(s):**

Cyclooxygenases (COXs) are known to play some roles in physiological mechanisms related to learning and memory. Since sodium salicylate is an inhibitor of COX, we have evaluated the effect of peripheral and intra-hippocampal administration of sodium salicylate on spatial learning and memory in male rats.

**Materials and Methods:**

Male rats were studied in two groups; the first group received different intraperitoneal (i.p.) sodium salicylate doses (0, 200, 300, and 400 mg/kg) and the second group received intra-hippocampal doses of the drug (0, 30, 50, and 100 μg/0.5 μl/side). The spatial performance of rats was tested using Morris water maze (MWM) task. The spatial learning and memory parameters were analyzed using ANOVA.

**Results:**

Peripheral and intra-hippocampal administration of sodium salicylate did not lead to a statistically significant change in the mean time (escape latency), and also the distance traveled for finding the hidden platform during the training days, compared with the control group. But at the probe trial, the percentage of time spent in the target quadrant by rats which received the highest doses of drug significantly increased.

**Conclusion:**

We found that both peripheral and intra-hippocampal administration of sodium salicylate facilitates the process of spatial memory consolidation in the MWM.

## Introduction

Prostaglandins are well-known metabolites of arachidonic acid (1), and their formation is catalyzed by cyclooxygenase (COX) enzymes (2). So far, two isoforms of COX enzymes have been identified; COX-1 and COX-2 (3, 4). COX-1 is the common form (5), while COX-2 is the induced form of the enzyme (6). The two forms of the enzyme can be found in different brain regions, including temporal cortex, amygdala, dentate gyrus, and hippocampus (7). The level of COX-2 expression in neurons is regulated by synaptic activities (8).

Non-steroidal anti-inflammatory drugs (NSAIDs) comprise a large family of medications that reduce production of prostaglandins by inhibiting different types of COXs (9), and their analgesic, antipyretic, and anti-inflammatory effects are well known (10). Administration of NSAIDs has a positive effect on prevention and healing of brain lesions resulted from overstimulation (11). Due to their low price, salicylates such as aspirin and sodium salicylate are still the most widely used drugs of the family (10, 12). Salicylates, but not indomethacine, have a neuroprotective effect in brain injuries induced by ischemia (13, 14). Salicylates also influence the GABA transports and exert great effects on ion channels, including N-methyl-D-aspartate (NMDA), long-acting calcium channels, and voltage-dependent sodium channels (15-17). Moreover, the COX-2 plays some roles in the activities related to neuronal plasticity (18). Therefore, it can be expected that NSAIDs, including salicylates can affect the learning and memory (19-21). Accordingly, it has been reported that intra-hippocampal injection of COX-2 inhibitors reduces memory consolidation (9). On the other hand, expression of COX-2 is increased in the cortex and hippocampus of patients affected by senile memory abnormalities (22). Jonker *et al* showed that acetylsalicylic improves the cognitive performance, including short-term memory, in the healthy elderly (23). Similar effects were observed with NSAIDs such as rofecoxib (selective COX-2 inhibitor) and naproxen (non-selective COX inhibitor) (24). However, there are few studies available on the protective effects of short-term use of NSAIDs on memory impairment (25). Furthermore, there are controversial findings with regards to the effects of COX inhibition on the learning process. For instance, Bruce Jones *et al* (1994) found that COX inhibition has reinforcing effects on memory (26), while Holscher (1995) reported impairing effects of COX inhibition indicate that the role of COX in learning and memory processes is more complex than what has been understood so far (19). Therefore, the study was carried out for the first time for evaluation of the simultaneous administration of peripheral and intra-hippocampal salicylate, as one of the most studied NSAIDs, on spatial learning and memory of young healthy rats using the Morris water maze.

## Materials and Methods

***In vivo experiments***

All experiments were carried out according to the National Institute of Health guide for the care and use of the laboratory animals, and approved by a local Animal Ethics Committee. All efforts were made to minimize the number of animals used and their suffering. Male Wistar rats (Razi institute, Tehran, Islamic Republic of Iran) aged 3-4 months and weighting 250-350 g, at the beginning of the experiments, were used. The rats were kept in animal facility room (20-22 °C) on the 12 hr light/dark cycle (lights on at 08:00 a.m.). Each 2-3 animals were housed in a 24X24X45 cm^3^ transparent, plastic cage. Except for the time of experiment, the animals had access to food and water *ad libitum*. Finally, from each group, 7-8 rats were used in the study.

***Experimental groups***

The animals were randomly divided into two groups. Animals in each group, that is, peripheral (PS) or central (CS) salicylate groups where then divided into four subgroups of 7-8 rats each.

Animals in the first group received sodium salicylate (0, 200, 300 or 400 mg/kg, i.p.). For the second group, we injected sodium salicylate (0, 30, 50 or 100 μg/ 0.5 μl/ side) into the hippocampal CA1 area. 

***Surgery and cannulation***

For drug injection into the CA1 region of the animals’ hippocamps, two cannulae were stereotaxically bilaterally implanted into hippocampal CA1 area. With this aim, the animals were anesthetized using ketamine (30 mg/kg, i.p.) and xylazine (2.5 mg/kg, i.p.) (27). Then, the rats were placed in the stereotaxic instrument and their heads were shaved, the guide cannulae were bilaterally placed 0.7 mm above the hipoocampal CA1 area using a needle of 22 gauges. The task was carried out consistent with the atlas of Paxinos and Watson (28) (from bregma: anteroposterior, −3.8 mm; mediolateral, ±2.2 mm; and 2.5 mm of the surface of skull). The cannulae were fixed on the skull surface using dentistry cement and a glasses screw. During the experiment, twenty min prior to the performing the test in the Morris water maze, the injection was performed through the guide cannulae, using a one µl Hamilton syringe, which was connected to an injection cannula. The injection cannula was made from a 27 dentistry gauge needle, which was cut in a way that 0.7 mm of the tip of the needle could come out and the drug could be easily injected and distributed in the desirable site. The syringe was kept at the injection site for 30 seconds after injection to help the liquid penetrate completely into the tissue spaces. The syringe was, then, slowly removed.

***Behavioral Assessment***

*Morris Water Maze (MWM) Apparatus*

The Morris water maze (MWM) consisted of a dark, circular pool of 140 cm in diameter and 70 cm in height. It was filled with 22±2 ^o^C water up to a height of 35 cm. A transparent plexiglas escape platform of 10 cm in diameter (indicated by the pilot study to be invisible to rats) was placed below water surface. The apparatus was located in a room with numerous extramaze cues that remained unchanged throughout the experiment. The time (escape latency), the distance swum to the platform (swim length), the swimming speed and the time spent in each quadrant were recorded using a video tracking system.

*Procedure*

The MWM procedure was conducted as described by Pourmotabbed *et al* (29). On the first day, the rats were placed on the escape platform in the middle of an empty pool for a brief duration of 60 seconds. The same procedure was repeated next day with the pool filled with water. If a rat should climb off the platform, it would be directed back onto it. Training started on the third day, with the platform placed in the center of the northwest quadrant. All rats experienced a daily session of four trials for six consecutive days. During each trial, rats were placed in the water while facing the pool wall at one of the four randomly determined starting points – north, west, east or south poles. Once a rat reached the platform, it was allowed to stay on it for 30 sec If a rat failed to find the platform within 60 sec, it would be directed to it and allowed to stay on it for 30 sec Subsequently, the rats were returned to their heated cages for a 30 s inter-trial period. Twenty four hr after the final training trial, spatial memory was examined with a probe trial. For this purpose, the platform was removed from the pool and the rats were allowed to swim about freely for 60 sec. The time spent in the quadrant which formerly contained the platform was recorded. In order to investigate whether any motivational factor interfered with the rats’ ability to escape, another trial was organized 24 hr after the probe trial; here, a visible platform was used and escape was guided by proximal, rather than distal, spatial cues. In this trial, the platform was raised above water surface and placed in the southeast quadrant while extramaze cues were removed from walls. The rats were allowed 60 sec to swim freely. The escape latency and swimming speed of the animals were recorded (29). 

***Tissue confirmation***

At the end of the experiment, 2 µl of methylene blue was injected in each cannula. Then, each rat underwent deep anesthesia to the death, its brain was removed and after fixation in formalin 10%, the brains were sectioned and dyed for confirmation of the injection site. Only the results obtained from the rats with confirmed cannula site were used in statistical analysis.

***Statistical analysis***

All data are presented as mean±SEM Significance of the escape latency and distance to platform (swim length) were assessed by two-way analysis of variance (ANOVA) with repeated measures and followed by Tukey’s test for multiple comparisons. Values for the probe trial were compared by one-way ANOVA and followed by Tukey’s test for multiple comparisons. Differences were considered significant at the level of *P*< 0.05 for all tests. 

## Results

***Evaluation of learning process during training days***

**Figure 1:**
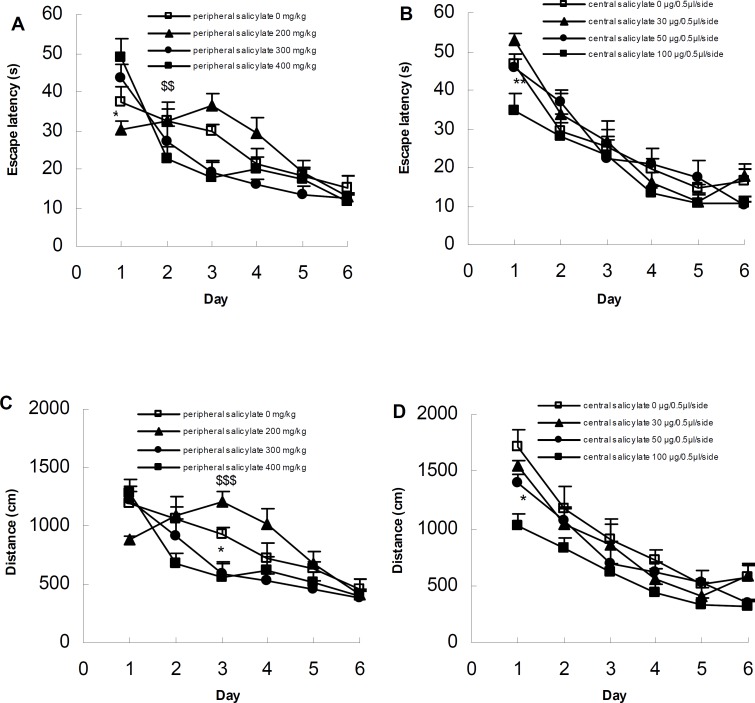
Evaluation of the effect of intra-peritoneal or intra-hippocampal administration of sodium salicylate on the learning trend in Morris water maze. The diagrams represent the escape latency (A, B) and the swimming distance (C, D) (Mean± SD) in the PS and CS subgroups over the training days (n= 7-8). (A) on the first training day, the escape latency in the group that received low dose intra-peritoneal drug (200 mg/kg) was significantly shorter than that in the group received high dose of the drug (400 mg/kg) (*P*<0.05). However, on the third training day, the escape latency time in the group that received a low dose of the drug (200 mg/kg) significantly increased, compared with the groups that received higher doses (300 and 400 mg/kg) ($$*P*< 0.01). (B) Only on the first training day, there was a significant decrease in the escape latency in the group that received a high dose of intra-hippocampal salicylate (100 μq/0.5 μl/side) compared with the CS subgroup that received a low dose of the drug (30 μq/0.5 μl/side) (**P< 0.01). (C) On the third training day and with regards to the swimming distance, there is a significant decrease in the group that received an intra-peritoneal high dose of the drug (400 mg/kg) compared with the control group (*P< 0.05). Moreover, on the third day of the training the distance was significantly shorter in the group received an intra-peritoneal low dose of the drug (200 mg/kg) compared with higher doses (300 and 400 mg/kg) ($$$*P*< 0.001). (D) Only on the first day of the training, the distance was significantly shorter in the group that received a high dose of intra-hippocampus (100 μq/0.5 μl/side) compared with other groups (*P< 0.05).

The MWM was used to assess the spatial performance. As shown in Figure 1, both PS and CS groups of rats learned to find the hidden platform and escape onto it.

Escape latency decreased over the six testing days in all PS subgroups (PS_0_, F_5,35_= 8.59, *P*< 0.0001; PS_200_, F_5, 35_= 10.35, *P*< 0.00001; PS_300_, F_5, 35_= 21.93, *P*= 0.0; PS_400_, F_5, 30_= 16.07, *P*= 0.0). A two-way repeated ANOVA applied to escape latencies of PS subgroups of rats found a significant day effect (F_5,135_= 43.72, *P*= 0.0), and also group and day interaction (F _5,135_= 4.1739, *P*= 0.0), but no group effect (F_ 3, 27_= 1.63, *P*= 0.20). The *post hoc *comparison indicated that the escape latency was significantly shorter on the first training day at the low dose of the drug (200 mg/kg), compared with the high dose (400 mg/kg) (*P*< 0.05). However, on the 5th day, a significant increase in the escape latency was observed in low dose of the drug (200 mg/kg), compared with the higher doses (300 and 400 mg/kg) (*P*< 0.01) (Figure 1A).

Moreover, evaluation of the results obtained in the CS subgroups showed that the escape latency decreased over the training days, which implies the positive trend of learning in these subgroups (CS_0_, F_5,25_= 14.74, *P*< 0.00001; CS_30_, F _5,30_= 26.33, *P*= 0.0; CS_50_, F_5,35_= 16.97, *P*= 0.0; CS_100_, F_5,35_= 13.35, *P*= 0.0). Statistical analysis of the results indicates that intra-hippocampal salicylate administration did not have a significant effect on the learning trend. Although the difference among the training days were statistically significant (F_5,125_= 65.81, *P*= 0.0), the difference among the groups (F_3,25_= 1.85, *P*= 0.16) and the interaction of groups and days (F_15,125_ = 1.47, *P*= 0.12) were not statistically significant. *Post hoc* comparison showed that there was a statistically significant difference between the subgroup that received low dose (30 μg/ 0.5 μl/side) and that received the high dose of the drug (100 μg/ 0.5 μl/side) only on the first day (*P*< 0.01) (Figure 1B).

When we tested the distance swum to the platform, we found that swim length decreased over training days in all PS subgroups, indicating the positive learning trend in the animals (PS_0_, F_5,35_= 7.92, *P*< 0.001; PS_200_, F_5,35_ = 11.65, *P*= 0.0; PS_300_, F_5,35_= 16.06, *P*= 0.0; PS_400_, F_5,30_= 13.16, *P*= 0.0). In general, the analysis of results showed significant difference among the studied groups (F_3,27_= 3.14, *P*< 0.05), training days (F_5,135_= 34.84, *P*= 0.0), and interaction of groups and days (F_5,135_= 3.88, *P*= 0.0). According to the *post hoc* comparison, the difference between the PS_0_ and PS_400_ subgroups was significant on the 3rd day of the experiment (*P*< 0.05). Also, comparison of the results showed that during the training days in lower dose of the drug (200 mg/kg), the changes in the swimming distance were similar to the changes in the escape latency time; such that up to day three, the learning gradually decreased and then it returned to the normal level. The *post hoc *comparison of the data demonstrated that on the third day, the distance swum by the PS_200_ subgroup was longer than those in the PS subgroups that received higher doses (*P*< 0.001) (Figure 1C).

Also, analysis of the results indicate that over the training days, the swimming distance decreased in the CS subgroups (CS_0_, F_5,25_= 11.50, *P*= 0.0; CS_30_, F_5,30_= 20.31, *P*= 0.0; CS_50_, F_5,35_= 15.93, *P*= 0.0; CS_100_, F_5,35_= 16.69, *P*= 0.0). A two-way repeated ANOVA applied to swim lengths of CS subgroups rats found a significant group (F_3,25_= 4.56, *P*< 0.05) and days (F _5,125_= 61.23, *P*= 0.0) effects, but no groups and days interaction (F _15,125_ = 1.04, *P*= 0.4). *Post hoc* analysis indicated that only on the first training day, there was a significant difference between the subgroup receiving high dose of the drug (100 μg/0.5μl) on one hand, and the other subgroups, on the other (*P*< 0.05) (Figure 1D).

***Evaluation of the memory consolidation process during probe trial***

**Figure 2:**
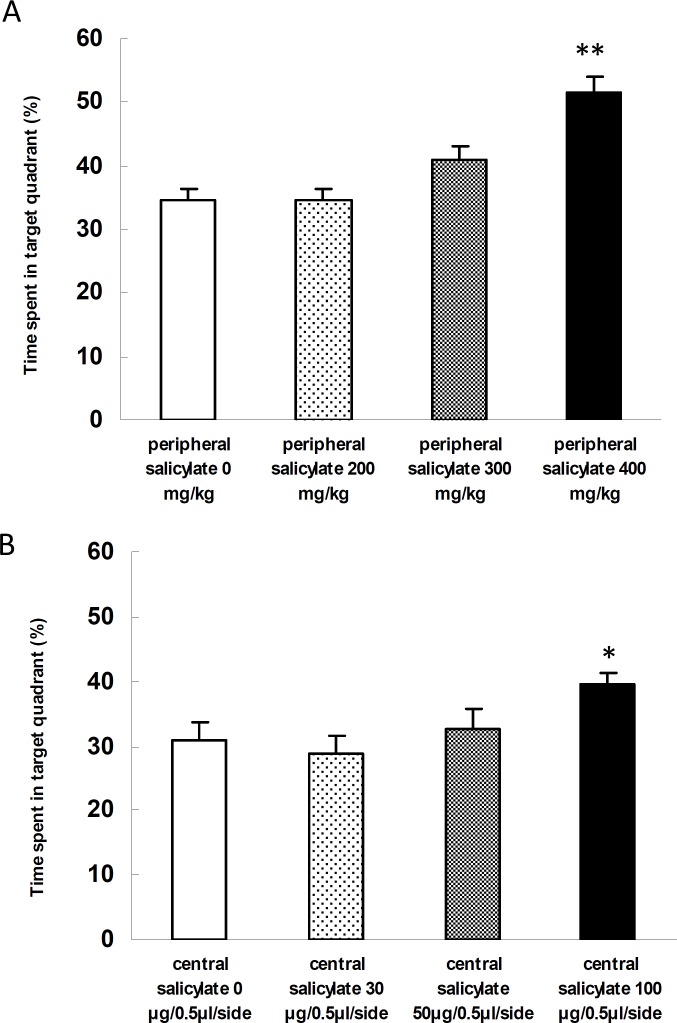
Evaluation of the effect of intra-peritoneal and intra-hippocampal administration of sodium salicylate on the process of memory consolidation during the probe trial. The diagrams compare the percentage of presence of animals in target quadrant (Mean percentage±SEM) in the groups receiving intra-peritoneal and intra-hippocampal salicylate (n= 7-8). (A) The presence of animals that received an intra-peritoneal high dose of the drug (400 mg/kg) in the target quarter was significantly higher than that in other groups (**P <0.01). (B) Only the difference between the group that received an intra-hippocampal high dose of the drug (100 μg/0.5 μl/side) and the group that received an intra-hippocampal low dose of the drug (30 μg/0.5 μl/side) was significant (*P <0.05).

In our experiments spatial memory formation was measured by the percentage of time swam in the target quadrant during probe trial. The probe test result is compared in Figure 2. When the performance of PS animals were tested, we found that the time spent in the target quadrant by the PS subgroups was different (F _3,27_ = 15.12 , *P*= 0.0). The difference was significant between the subgroup received 400 mg/kg on one hand, and the other subgroups on the other (*P*< 0.01) (Figure 2A).

Also, the results indicated that the CS subgroups were different with respect to the percentage of presence of the rats in the target quadrants (F _3,25_ = 3.12 , *P*< 0.05). The difference between the subgroups that received 30 μg/0.5 μl/side and that received 100 μg/0.5 μl/side was statistically significant (*P*< 0.05) (Figure 2.B).

However, comparing performance of different groups on a visible platform trial showed no significant differences in swim lengths and speeds (*P* > 0.05) (data not shown).

## Discussion

According to the results, the peripheral or intra-hippocampal administration of sodium salicylate did not have a significant effect on the trend of spatial learning, but it facilitated the consolidation of spatial memory. Previous studies demonstrated that COX inhibitors prevent memory impairment resulting from advanced ages (23, 24). Accordingly, NS-398 (a selective COX-2 inhibitor) reduced the detrimental effects of hypoxia on spatial memory (30) and indomethacine (as a non- selective COX inhibitor) prevented the memory impairment resulting from non-inflammatory processes (22).

In spite of our results, there are controversial results in the literature. Teather *et al* reported that peripheral administration of indomethacin and NS-398 impaired spatial learning and memory in the MWM via reduced production of prostaglandins (31). Prostaglandins are major glutaminergic (32), adrenergic, and noradrenergic (33) transmission modulators. As at the beginning of the learning process the activity of NMDA receptors increases, the concentration of prostaglandins increases (34), too, which in turn plays an important role in transmission of messengers related to memory process (35, 36). It should be noted that piroxicam (as a non-selective COX-1 inhibitor) did not have an impairing effect on memory (24), which can be due to the different roles it plays in the learning process (31, 37). In this respect, high doses of acetaminophen can lead to spatial learning impairment, while lower doses can facilitate spatial performance in the MWM (38).

In a part of our study, a dual effect of peripheral low dose salicylate administration (200 mg/kg) was observed. We found that in the early days of the experiment, the escape latency and the swim length increased in the group. After some days, the inhibitory effect was removed and learning was normalized. It can be proposed that learning impairment in the early days of the experiment resulted from the detrimental effect of salicylate on learning and after some days, the stimulatory effect of salicylate became dominant and therefore, the learning trend was normalized. In higher salicylate doses, the simultaneous occurrence of facilitatory and inhibitory effects of salicylate prevents the occurrence of inhibitory effect of the drug in the learning trend.

We also evaluated the effect of the intra-hippocampal administration of salicylate in young healthy animals for the first time. The results indicated that administration of drug did not lead to a significant effect on spatial learning, but during the probe trial, administration of high dose ended in a significant increase in percentage of presence of the animals in the target quadrants, compared with that of the low dose. Previous studies indicated that although intra-hippocampal administration of a selective COX-2 inhibitor (celecoxib) resulted in impairment of spatial memory in the MWM, injection of a non-selective COX inhibitor (indomethacin) did not lead to a significant effect on spatial memory. The difference was attributed to the different effects of COX-1 and COX-2 on memory process (9, 39). 

Other studies demonstrated that COX pathway is one of the regulatory factors in GABA-ergic transmission in the CNS (40), and in the presence of sodium salicylate, the rate of GABA secretion (41) and GABA_A_ receptor-mediated responses decreased significantly. Therefore, by pre-synaptic GABA inhibition, sodium salicylate increases the excitability of pyramidal neurons of hippocampus (42-44) which led to increased excitability of neurons involving in memory formation. Furthermore, sodium salicylate increases the number and activity of NMDA receptors (15, 21) involved in the processes of learning and memory (45). Moreover, sodium salicylate prevents the overexpression of COX-2 (46). Long-term presence of COX-2 inhibitory factors in the dentate gyrus reduces the incidence of LTP occurrence in the region (47). Also, prostaglandins can reverse the detrimental effect of COX-2 inhibitors on learning and memory (48). The reducing effect of COX-2 inhibitors is probably a product of the decreased cholinergic transmission in the presence of the inhibitors (39). Hence, it can be mentioned that salicylate has both stimulatory and inhibitory effects on hippocampus. The stimulatory effect is caused by the decreased GABA-ergic inhibitory activity and increased glutaminergic stimulatory effect. The inhibitory effect resulted from the decreased prostaglandin function and cholinergic transmission. If the stimulatory and inhibitory effects of salicylates occur simultaneously and fairly with the same strength in hippocampus, the overall effect on the memory formation is almost neutral. This can partly justify the observations of the present study with regard to lack of effect of intra-hippocampal injection of low doses of salicylate on spatial learning and memory. Accordingly, high dose injection of salicylate into the CA1 region of hippocampus leads to dominance of stimulatory effect of the drug on its inhibitory effect, and consequently facilitation of retrieval of spatial memory. The available results on the dual effect of peripheral administration of a low dose of salicylate (200 mg/kg) can be another supportive reason for the simultaneous stimulatory and inhibitory effects of the drug on learning and memory. This can also explain the ineffectiveness of central administration of low doses of salicylate on learning and memory. However, evaluation of the role of each of the mechanisms requires further studies.

We also demonstrated that peripheral and central administration of salicylate did not lead to a significant effect on the escape latency and also on the swimming speed on a visible platform trial. Thus, it can be mentioned that salicylate does not have a negative effect on visual and sensory-motor systems of the animals, and the results obtained are related to the processes that take place during the spatial learning.

## Conclusion

The results demonstrated that peripheral administration of sodium salicylate improved the process of spatial memory consolidation in Morris water maze. Moreover, injection of a high dose of drug into the CA1 region of hippocampus can lead to a similar result. The mechanisms involving in the phenomena are not known and require further studies.
